# MET fusions are targetable genomic variants in the treatment of advanced malignancies

**DOI:** 10.1186/s12964-023-01454-0

**Published:** 2024-01-09

**Authors:** Dantong Sun, Xiaoming Xing, Yongjie Wang, Helei Hou

**Affiliations:** 1 Department of Medical Oncology, National Cancer Center/National Clinical Research Center for Cancer/Cancer Hospital, Chinese Academy of Medical Sciences and Peking Union Medical College, Beijing, 100021 China; 2 State Key Laboratory of Molecular Oncology, National Cancer Center/National Clinical Research Center for Cancer/Cancer Hospital, Chinese Academy of Medical Sciences and Peking Union Medical College, Beijing, 100021 China; 3 Department of Pathology, The Affiliated Hospital of Qingdao University, Qingdao, 266000 Shandong China; 4 Department of Thoracic Surgery, The Affiliation Hospital of Qingdao University, No. 59 Haier Road, Qingdao, 266000 Shandong China; 5 Department of Oncology, The Affiliated Hospital of Qingdao University, No. 7 Jiaxing Road, Qingdao, 266000 Shandong China

**Keywords:** Cancer, *MET* fusions, MET-TKIs, Targeted therapy

## Abstract

Targeted therapy for malignancies has developed rapidly in recent years, benefiting patients harboring genetic mutations sensitive to relevant tyrosine kinase inhibitors (TKIs). With the development of targeted sequencing techniques, an increasing number of detectable genomic alterations in malignancies, including *MET* fusions, have been revealed. *MET* fusions, although rare among malignancies, might be functional driver genes that participate in activating downstream signaling pathways and promoting cell proliferation. Therefore, it is believed that *MET* fusions could be targetable genomic variants of *MET*, and inhibition of *MET* is considered an optionable therapeutic choice for patients harboring *MET* fusions. According to the summary presented in this review, we recommend MET-TKIs as suitable treatment agents for patients harboring primary *MET* fusions. For patients harboring acquired *MET* fusions after the development of resistance to TKIs targeting primary genomic alterations, such as sensitive *EGFR* mutations, treatment with a MET-TKI alone or in combination with TKIs targeting primary genomic alterations, such as EGFR-TKIs, is hypothesized to be a reasonable option for salvage treatment. In summary, *MET* fusions, despite their low incidence, should be taken into consideration when developing treatment strategies for cancer patients.

## Introduction

Currently, the high morbidity and mortality of malignancies result in serious public health problems, especially in China. According to the latest report, the total number of cancer-related deaths in 2014 was 2,205,200 (1,425,700 men and 779,500 women), accounting for 22.40% of all deaths in 2014 in China [1]. Given this critical situation, various therapeutic approaches, especially targeted therapies for patients harboring sensitive genomic alterations, have developed rapidly in the past decade. Tyrosine kinase inhibitors (TKIs) are the best representatives of targeted therapeutic agents, and they provide more opportunities for therapeutic success and benefit cancer patients. For instance, TKIs targeting sensitive mutations in *epidermal growth factor receptor* (*EGFR*), such as gefitinib, erlotinib and osimertinib, prolong progression-free survival (PFS) and overall survival (OS) in patients with non-small cell lung cancer (NSCLC), with acceptable adverse events during treatment [2]. Anaplastic lymphoma kinase (*ALK*) gene rearrangement is a well-known genomic mutation that can be effectively treated with targeted therapy. Patients with NSCLC who have this *ALK* rearrangement display significant responsiveness to specific inhibitors, such as crizotinib and alectinib [3].. Inhibitors targeting gene fusions involving *neurotrophin receptor kinase* (*NTRK*) have demonstrated efficacy in several cancer types, including NSCLC [4]. With the development of molecular detection techniques and the increase in the detection depth of targeted sequencing, an increasing number of novel genomic alterations have become detectable and targetable in anticancer treatment.

A series of studies revealed solid tumors with oncogenic addiction to *MET* alterations and elucidated the efficacy of MET-TKIs in the treatment of patients harboring sensitive *MET* alterations. The *MET* gene is located on chromosome 7 and encodes the MET protein. The MET protein was demonstrated to be the receptor for hepatocyte growth factor (HGF), which participates in the biological regulation of cell proliferation [5]. In addition, *MET* alterations are suggested to be one of the major uncommon oncogenic alterations in NSCLC [5], and the latest research identified the important role of *MET* amplification in accelerating the growth of tumors and predicting the poor prognosis of patients with malignancies [6, 7]. *MET* amplification and other oncogenic mutations, such as *MET* exon 14 skipping mutations, can activate signal transduction in carcinogenic pathways belonging to MET signaling [8]. According to the results of clinical trials for patients harboring these *MET* alterations, MET-TKIs are believed to be an efficient and safe choice for these patients. Capmatinib and tepotinib, which benefit patients with metastatic NSCLC with *MET* exon 14 skipping mutations, were the first two MET-TKIs approved by the US Food and Drug Administration [9]. Moreover, the anticancer efficacy of savolitinib, another MET-TKI, for the treatment of advanced NSCLC patients harboring *MET* exon 14 skipping mutations was confirmed in a phase 2 study [10]. Crizotinib, which targets *ALK*, *ROS1* and *MET*, could also constitute a treatment option for patients harboring sensitive *MET* alterations.

*MET* fusions were reported in recent studies as novel detectable alterations of *MET*, although the incidence of *MET* fusions is low [11–17]. It is likely that patients harboring *MET* fusions might respond to specific treatments, such as crizotinib, as reported; however, the demographic characteristics and treatment data have not been well reported. In this review, we determine the incidence rates of *MET* fusions and the demographic characteristics of the affected patients through online databases and published studies. In addition, we summarize the cellular oncogenic functions of *MET* fusions based on previous studies. We also include reported cancer patients harboring primary or acquired *MET* fusions to understand the potential treatment options for these patients. Our findings suggest that *MET* fusions, despite their low incidence, should be considered when developing treatment strategies for cancer patients.

### Identification of *MET* fusions in malignancies: incidence rates, demographic characteristics and cellular oncogenic functions

Even though genetic changes in the MET gene are quite prevalent in certain types of tumors, particularly in melanoma and NSCLC, where more than 5% of patients have MET gene alterations [18], the incidence of *MET* fusions is still low in all cancer types. According to the latest studies, less than 1.1% of patients harbor *MET* fusions [18–20]. The incidence of *MET* fusions was the highest in brain cancer, in which it was detected in approximately 1.10% of patients, followed by bile duct cancer (0.52% of patients), lung cancer (0.07–0.30% of patients), gastric cancer (0.25% of patients) and intestinal cancer (0.14% of patients). Fewer than 0.10% of patients with other cancer types harbor *MET* fusions, according to published studies.

**Fig. 1:**
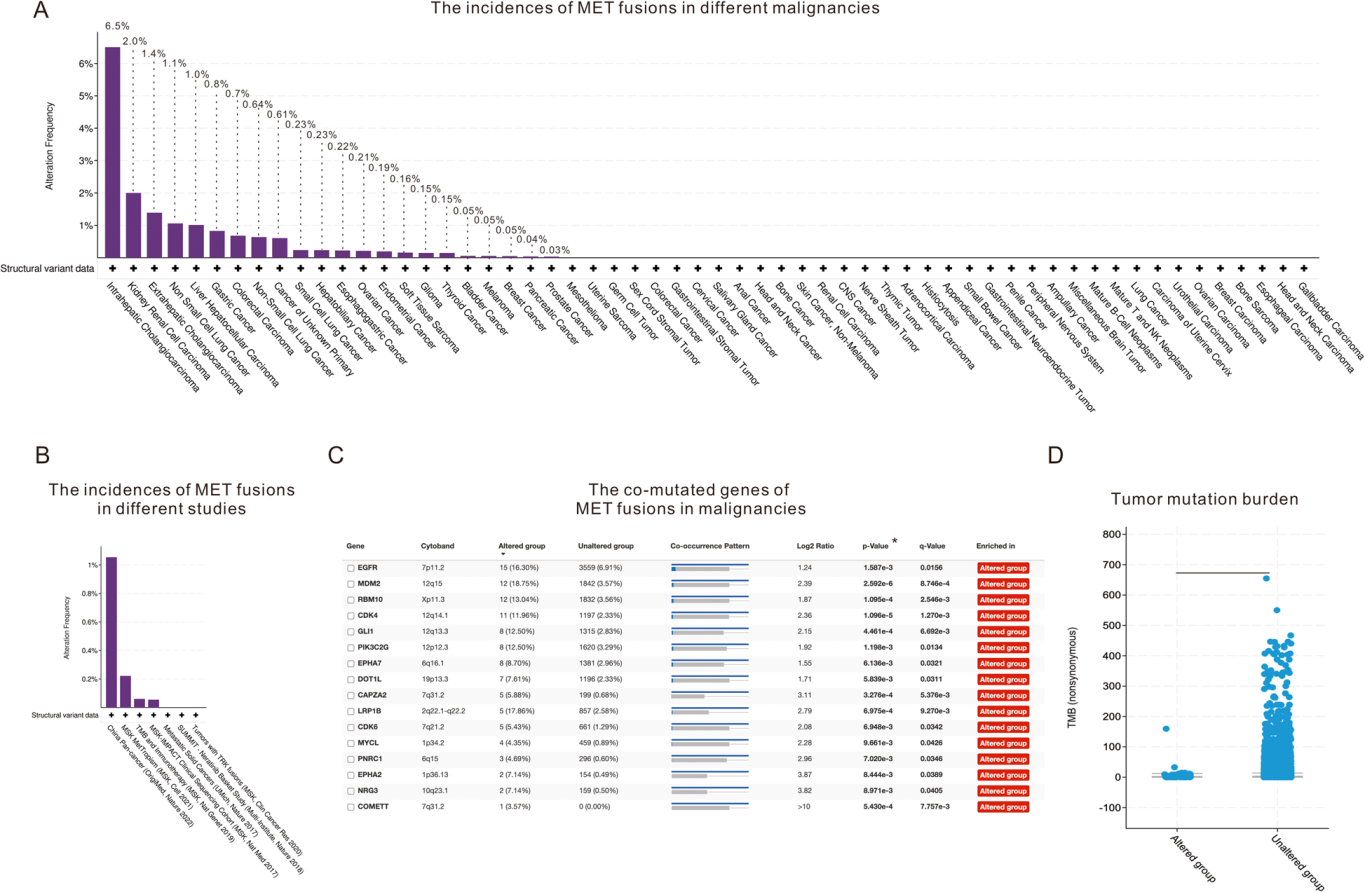
The prevalence and molecular characteristics of MET fusions in malignant tumors. A) Incidences of *MET* fusions in malignancies; only structural variant data were involved during the searching process, and only fusions were found in the MET structural variant data; B) the incidences of *MET* fusions in different studies; C) the comutated genes detected with *MET* fusions in malignancies; cytoband: cytogenetic bands; co-occurrence pattern: upper row refers to samples colored according to group, and lower row refers to samples with an alteration in the listed gene; D) the tumor mutation burden between patients with *MET* fusions or wild type of *MET* gene

To further explore the incidence rates of *MET* fusions in a larger sample of patients, we used the cBioPortal for Cancer Genomics database [21, 22] and analyzed the results. In total, 75,661 patients with different cancer types in 10 pancancer studies were included in our analysis, and the incidence of *MET* fusions increased with increasing sample size in each type of cancer. The results showed that 6.50% of intrahepatic cholangiocarcinoma patients, 0–2.00% of renal cell carcinoma patients, 1.39% of extrahepatic cholangiocarcinoma patients, 0.64–1.06% of NSCLC patients, 1.01% of hepatocellular carcinoma patients, 0.23% of small cell lung cancer (SCLC) patients, 0.23% of hepatobiliary cancer patients, 0.22% of esophagogastric cancer patients, 0.21% of ovarian cancer patients, 0.19% of endometrial cancer patients, 0.15% of soft tissue sarcoma patients, 0.15% of glioma patients and 0.15% of thyroid cancer patients harbored detectable *MET* fusions as shown in Fig. 1A. The prevalence of MET fusions may be relatively low, but considering the large number of cancer patients, there is still a substantial population at risk. Hence, it is still meaningful to outline the traits of patients with MET fusions and to offer treatment choices for these individuals. Additionally, it is noteworthy that gene fusion detection is technically challenging. The use of various genomic examination methods in different datasets might cause inaccurate analysis based on current open-access data.

According to the cBioPortal for Cancer Genomics database, less than 1% (92/75661) of patients harbor detectable *MET* fusions. However, whether there are differences between races is unclear. As shown in Fig. 1B, Asian patients (China Pan-Cancer, OrigiMed, Nature 2022) had the highest incidence of *MET* fusions, at approximately 1.05%, which was much higher than the second highest incidence (MSK MetTropism, MSK, Cell 2021, incidence of *MET* fusions: 0.22%). To the best of our knowledge, only two Chinese studies [18, 19] have reported the demographic characteristics of lung cancer patients harboring *MET* fusions. These studies demonstrated that the age of patients was not a specific factor that influenced the fusion of genes and that patients between 27 and 83 years old harbored detectable *MET* fusions. The two studies noted that most of the lung cancer patients (89.3–92.3%) harboring *MET* fusions were diagnosed with lung adenocarcinoma (LUAD). However, the smoking history of patients was not clearly related to *MET* fusions, according to the two studies. In this review, we summarize the reported demographic characteristics of cancer patients harboring *MET* fusions. The selection criteria for this review is as follows: 1. Patient population: patients with malignant tumors identified as carrying MET fusion in reviews, case reports, or studies; 2. Intervention measures: inclusion of patients without restriction on the type of treatment, but with a focus on collecting data on those who have undergone MET-TKI treatment; 3. Search terms: MET, MET fusion, MET fusions, MET mutations, and MET alterations in PubMed; Period of search: August 2023 to September 2023. As concluded from the studies [11–19, 23–32] listed in Table 1, the average age of patients diagnosed with malignancies harboring *MET* fusions was 56.6 years, with a range of 27 to 74 years. Among these patients, 60% patients were female, and the *MET* fusion incidence showed no significant sex difference.

**Table 1:** Demographic characteristics and clinical information of patients harboring MET fusions

References	Case	Age	Sex	Organ origin	Histology diagnosis	Stage	Test methods	Sample type	Detection period	MET fusion	MET amplification	MET exon 14 skipping mutation	MET-TKIs treatment (Response, PFS)
Rooper LM, et al. Am J Surg Pathol. 2018 [29]	1	59	Female	Salivary gland	ACC	III	NGS	FFPE	Primary	ETV6-MET	–	–	1 L Cabozantinib (PR, PFS greater than 1.0 m)
Cho JH, et al. J Thorac Oncol. 2018 [11].	2	51	Female	Lung	ADC	IV	NGS	NA	Primary	KIF5B-MET	–	–	4 L Crizotinib(PR, PFS 10 m)
Liu J, et al. J Thorac Oncol. 2019 [12].	3	60	Female	Lung	ADC	IV	NGS	FFPE	Acquired (EGFR)	CAV1-MET	–	–	1 L Crizotinib+Osimertinib (PR, PFS greater than 2.0 m)
Blanc-Durand F, et al. Oncologist. 2020 [16].	4	43	Female	Lung	ADC	IV	NGS	NA	Primary	HLA-DRB1-MET	–	–	2 L Crizotinib (CR of lung/brain nodules, 7.0 m); 3 L Tepotinib (CR of lung/brain nodules, 10.0 m); 4 L Cabozantinib (SD, greater that 5.0 m)
Yu Y, et al. Oncologist. 2020 [27].	5	41	Female	Bile duct	ICC	IV	NGS	FFPE	Primary	EHBP1-MET	–	–	1 L Crizotinib (PR, PFS 8.0 m)
Wu ZW, et al. World J Clin Cases. 2021 [28].	6	64	Male	Stomach	ADC	IV	NGS	FFPE	Primary	KIF5B-MET	–	–	NA
Liu LF, et al. World J Clin Cases. 2022 [15].	7	59	Female	Lung	ADC	IV	NGS	FFPE	Primary	HLA-DRB1-MET	–	–	2 L Crizotinib (CR, PFS greater than 10.0 m)
Ma Q, et al. Front Oncol. 2022 [14].	8	60	Female	Lung	ADC	IV	NGS	FFPE	Primary	ARL1-MET	–	–	1 L Crizotinib (PR, PFS 5.0 m)
Ou L, et al. Front Oncol. 2022 [13].	9	63	Female	Lung	ADC	IV	NGS	FFPE	Acquired (EGFR)	CUX1-MET	–	–	4 L Crizotinib+Icotinib (PR, PFS 9.0 m)
Li Y, et al. Clin Lung Cancer. 2022 [17].	10	52	Female	Lung	ADC	IV	NGS	FFPE	Acquired (EGFR)	MET-DSTN	–	–	4 L Crizotinib+Gefitinib (CR, PFS greater than 6.0 m)
Lin CY, et al. Front Oncol. 2022 [25].	11	56	Female	Lung	ADC	III	NGS	NA	NA	KIF5B-MET	–	–	2 L Capmatinib (PR, PFS greater than 9.0 m)
Liu J, et al. Transl Cancer Res. 2022 [23].	12	72	Male	Lung	ADC	IV	NGS	FFPE	Primary	CD47-MET	–	–	1 L Crizotinib (PR, PFS 8.0 m); 2 L Cabozantinib (PR, PFS 3.0 m)
Liu LF, et al. World J Clin Cases. 2022 [15].	13	33	Female	Lung	ADC	IV	NGS	NA	Primary	KIF5B-MET	NA	NA	3 L Crizotinib (PR, PFS 8.0 m)
	14	62	Female	Lung	ADC	IV	NGS	NA	Primary	STARD3NL-MET	NA	NA	1 L Crizotinib (PR, PFS 14.0 m)
	15	56	Female	Lung	ADC	IV	NGS	FFPE	Primary	MET-ATXN7L1	–	–	2 L Crizotinib (PR, PFS 4.0 m)
	16	43	Female	Lung	ADC	IV	NGS	FFPE	Acquired (EGFR)	MET-UBE2H	–	–	2 L Crizotinib (PR, PFS 6.5 m)
Kang J, et al. Lung Cancer. 2023 [19].	17	52	Female	Lung	ADC	IV	NGS	FFPE	NA	MET-DST	–	+	1 L Capmatinib (PR, PFS 12.6 m)
	18	57	Female	Lung	ADC	IV	NGS	FFPE	NA	EPHB4-MET	–	–	1 L Crizotinib (PR, PFS 8.8 m), 2 L Cabozantinib (PD, PFS 1.0 m), 4 L PLB-1001 (PD, PFS 1.0 m)
	19	70	Male	Lung	ADC	IV	NGS	FFPE	NA	EPHB4-MET	+	–	1 L Savolitinib (PR, PFS greater than 7.5 m)
	20	73	Male	Lung	ADC	IV	NGS	FFPE	NA	CD74-MET	–	–	3 L Crizotinib (PR, PFS 7.0 m)
	21	40	Female	Lung	ADC	IV	NGS	FFPE	NA	CD47-MET	–	–	5 L Savolitinib (PR, PFS 6.5 m)
	22	41	Male	Lung	ADC	IV	NGS	FFPE	NA	CCDC6-MET	–	–	4 L Crizotinib (PR, PFS 5.0 m)
	23	61	Male	Lung	ADC	IV	NGS	FFPE	NA	HLA-DRB1-MET	–	–	3 L Ensartinib (SD, PFS 4.0 m)
	24	38	Female	Lung	ADC	IV	NGS	FFPE	NA	CD47-MET	+	–	3 L PLB1001 (SD, PFS 3.0 m)
	25	56	Male	Lung	SCLC	III	NGS	Plasma	NA	TNPO3-MET	–	–	1 L Crizotinib (PD, PFS 1.5 m)
	26	58	Female	Lung	ADC	IV	NGS	FFPE	NA	CD74-MET	–	–	5 L Crizotinib (PD, PFS 1.0 m)
	27	66	Male	Lung	ADC	IV	NGS	FFPE	NA	CAV1-MET	–	–	6 L Ensartinib (PD, PFS 1.0 m)
	28	55	Male	Lung	ADC	IV	NGS	FFPE	NA	KIF5B-MET	–	–	3 L Crizotinib (PD, PFS 1.0 m)
	29	64	Male	Lung	ADC	IV	NGS	FFPE	NA	THAP5-MET	–	–	NA
Sun D, et al. J Transl Med. 2023 [18].	30	66	Male	Lung	NSCLC	IV	NGS	FFPE	Primary	ECT2-MET	–	–	NA
	31	36	Female	Lung	ADC	I	NGS	FFPE	Primary	MET-EPHA1	–	–	NA
	32	71	Male	Lung	ADC	IV	NGS	FFPE	Primary	CD47-MET	–	–	NA
	33	48	Male	Lung	ADC	II	NGS	FFPE	Primary	MET-HLA-DRB5	–	–	NA
	34	49	Male	Lung	ADC	III	NGS	FFPE	Primary	MET-GJC2	–	–	NA
	35	68	Male	Lung	SCC	IV	NGS	Pleural effusion	Primary	WEE2-AS1-MET	–	–	NA
	36	52	Female	Lung	SCC	IV	NGS	FFPE	Primary	LRIG3-MET	–	–	NA
	37	47	Female	Lung	ADC	III	NGS	FFPE	Primary	CD47-MET	–	–	NA
	38	56	Female	Lung	ADC	IV	NGS	Fresh tissue	Primary	EPHB4-MET	–	–	NA
	39	57	Female	Lung	ADC	IV	NGS	FFPE	Primary	EPHB4-MET	–	–	NA
	40	68	Female	Lung	ADC	IV	NGS	Plasma	Primary	HLA-DRB1-MET	–	–	NA
	41	69	Male	Lung	ADC	IV	NGS	FFPE	Primary	MET-CTTNBP2	–	–	NA
	42	72	Male	Lung	SCLC	IV	NGS	FFPE	Primary	CAPZA2-MET	–	–	NA
	43	57	Male	Lung	ADC	IV	NGS	FFPE	Primary	MET-FOXP2	+	–	NA
	44	27	Male	Lung	ADC	IV	NGS	Plasma	Acquired (EGFR)	COG5-MET	+	–	NA
	45	57	Male	Lung	ADC	I	NGS	Plasma	Primary	CFTR-MET	–	–	NA
	46	68	Male	Lung	ADC	IV	NGS	Plasma	Primary	HLA-DRB1-MET	–	–	NA
	47	57	Female	Lung	ADC	IV	NGS	Plasma	Acquired (EGFR)	KCND2-MET	+	–	NA
	48	48	Female	Lung	ADC	IV	NGS	Plasma	Acquired (EGFR)	MET-ADAP1	–	–	NA
	49	63	Female	Lung	ADC	IV	NGS	Plasma	Primary	MET-LINC01392	–	–	NA
	50	70	Male	Lung	ADC	IV	NGS	FFPE	Primary	CTNNA3-MET	+	–	NA
	51	51	Female	Lung	ADC	IV	NGS	Plasma	Acquired (EGFR)	WNT2-MET	+	–	NA
	52	56	Male	Lung	ADC	IV	NGS	FFPE	Primary	KIF5B-MET	–	–	NA
	53	71	Male	Lung	ADC	IV	NGS	FFPE	Primary	CD47-MET	–	–	NA
	54	34	Female	Lung	ADC	IV	NGS	FFPE	Primary	ST7-MET	+	–	NA
	55	42	Female	Lung	ADC	III	NGS	Fresh tissue	Primary	MET-STEAP4	+	–	NA
	56	65	Female	Lung	ADC	IV	NGS	Pleural effusion	Acquired (EGFR)	MET-DOCK4	+	–	NA
	57	70	Female	Lung	ADC	III	NGS	Fresh tissue	Primary	TFEC-MET	–	–	NA
	58	52	Female	Lung	ADC	IV	NGS	Pleural effusion	Primary	MET-DST	–	+	NA
	59	61	Male	Lung	SCC	IV	NGS	FFPE	Primary	EML4-MET	–	–	4 L Crizotinib (PR, PFS greater than 1.0 m)
Yang Y, et al. Clin Lung Cancer. 2023 [24].	60	67	Female	Lung	ADC	III	NGS	FFPE	Primary	PRKAR1A-MET	–	–	1 L Crizotinib (PR, PFS 1.0 m, Stopped treatment because of AEs); 2 L Crizotinib (CR, PFS greater than 9.0 m)
Turpin A, et al. Oncologist. 2023 [26].	61	49	Male	Bile duct	ICC	IV	NA	NA	Primary	CAPZA2-MET	+	–	3 L Capmatinib (PR, PFS 4.0 m)
Riedel R, et al. J Thorac Onco. 2023 [30].	62	NA	NA	Lung	ADC	NA	NA	NA	NA	KIF5B-MET	–	NA	2 L tepotinib (PR, DOR 25.6 m)
	63	NA	NA	Lung	ADC	NA	NA	NA	NA	ST7-MET	+	NA	NA
	64	NA	NA	Lung	ADC	NA	NA	NA	NA	TRIM4-MET	–	NA	NA
	65	NA	NA	Lung	ADC	NA	NA	NA	NA	ST7-MET	+	NA	2 L tepotinib (PD)
	66	NA	NA	Lung	ADC	NA	NA	NA	NA	PRKAR2B-MET	+	NA	NA
	67	NA	NA	Lung	ADC	NA	NA	NA	NA	CAPZA2-MET	–	NA	NA
	68	NA	NA	Lung	ADC	NA	NA	NA	NA	CAPZA2-MET	–	NA	2 L tepotinib (PR, DOR 7 m)
	69	NA	NA	Lung	ADC	NA	NA	NA	NA	CAPZA2-MET	+	NA	NA
	70	NA	NA	Lung	ADC	NA	NA	NA	NA	CAPZA2-MET	–	NA	NA
Davies KD, et al. JCO Precis Oncol. 2017 [31].	71	74	Female	Lung	ADC	IV	NGS	NA	Primary	HLA-DRB1-MET	NA	–	1 L Crizotinib (CR, greater than 8.0 m)
Xia H, et al. Cancer Med. 2023 [32]	72	67	Female	Lung	NA	IV	NGS	NA	NA	HLA-DRB1-MET	NA	NA	5 L Crizotinib (PR, 12 m)
	73	49	Female	Lung	ADC	III	NGS	NA	Acquired (EGFR)	LINC01392-MET	+	NA	4 L Crizotinib+Osimertinib (PR, 3 m)
	74	62	Female	Lung	ADC	IV	NGS	NA	NA	KIF5B-MET	NA	NA	2 L Crizotinib (PD, 2 m)

Abbreviations: ACC: acinic cell carcinoma; ADC: adenocarcinoma; ICC: intrahepatic cholangiocarcinoma; NGS: next generation sequencing; FFPE: formalin fixed paraffin embedded; CR: complete response; PR: partial response; PD: progressive disease; SD: stable disease; PFS: progression-free survival; DOR, duration of response; NA: not available; “+”: positive genetic variation; “-”: negative genetic variation

As described in the introduction section, wild-type MET protein serves as the receptor for HGF and can be activated (phosphorylated) by stimulation with HGF. Multiple research studies have concentrated their efforts on investigating the impact of the MET fusion protein, a variant protein, on the cellular function of MET [19, 33–37]. The results suggested that *MET* fusions, such as *PTPRZ1–MET* in glioma and glioblastoma cells or *KIF5B-MET* and *EPHB4-MET* in lung cancer cells, can upregulate the expression and enhance the phosphorylation of the MET protein, even without HGF stimulation. In certain instances, particularly with *PTPRZ1-MET* fusions, the point where the *MET* gene is disrupted occurs before the start codon, as happens in the partner gene. As a result, the resulting protein from these fusions would likely be the complete MET protein, but now regulated by a potentially more active promoter [38]. The abnormally constitutively phosphorylated MET protein can then activate downstream signaling pathways, including the MAPK pathway, Akt pathway and STAT3 pathway, events characterized by the upregulated phosphorylation of ERK1/2, Akt and STAT3, respectively. The latter three pathways were all believed to be associated with tumor proliferation and apoptosis escape, and these hypotheses were verified in the listed studies [34, 35] through in vitro and in vivo experiments. Thus, these aberrant fusions of the *MET* gene act as a “driver” for the activation of the MET protein and downstream signaling pathways, endowing tumor cells with HGF-independent self-activation ability. Fortunately, current preclinical evidence in the listed studies suggested that tumor cells harboring *MET* fusions showed sensitivity to treatment with MET-TKIs, such as crizotinib, tepotinib, SGX523 and foretinib, indicating the basis for treatment methods for patients harboring *MET* fusions. Most importantly, *MET* fusions enable tumor cells to abolish their dependency on the ligand HGF and participate in the autophosphorylation of components in oncogenic cellular pathways. The oncogenic function of *MET* fusions makes them therapeutic targets for cancers.

### The molecular landscape of human genomes harboring *MET* fusions: detection methods, fusion partners, and comutated genes

As shown in Table 1, the next-generation sequencing (NGS) method was used for *MET* fusion detection in all reported patients, but the detection panels varied among patients. Both DNA-based and RNA-based NGS are considered optionable methods. RNA-based NGS sequencing is believed to be more sensitive and could widen the map of druggable targets, although it requires high sample quality [38]. The conventional samples used for NGS examination were all suitable for MET fusion detection. Most samples used for NGS were formalin-fixed paraffin-embedded (FFPE) samples (41/74, 55.4%). In addition, other types of samples, such as plasma (9/74, 12.2%), fresh tissues (3/74, 4.1%) and pleural effusions (3/74 4.1%), could all be used for *MET* fusion detection according to the cases in Table 1.

Regarding the types of *MET* gene fusion, in this review, we determined the incidence of each type of *MET* fusion and found that *KIF5B-MET* (8/74, 10.8%), *HLA-DRB1-MET* (8/74, 10.8%), *CAPZA2-MET* (6/74, 8.1%) and *CD47-MET* (6/74, 8.1%) were the four most common mutation types, followed by *EPHB4-MET* (4/74, 5.4%), *ST7-MET* (3/74, 4.1%), *CAV1-MET* (2/74, 2.7%), *CD74-MET* (2/74, 2.7%) and *MET-DST* (2/74, 2.7%). Other types of *MET* fusion, such as *MET-ADAP1*, *ARL1-MET*, *MET-ATXN7L1*, *CCDC6-MET*, *CFTR-MET*, *COG5-MET*, *CTNNA3-MET*, *MET-CTTNBP2*, *CUX1-MET*, *MET-DOCK4*, *MET-DSTN*, *ECT2-MET*, *EHBP1-MET*, *EML4-MET*, *MET-EPHA1*, *ETV6-MET*, *MET-FOXP2*, *MET-GJC2*, *MET-HLA-DRB5*, *KCND2-MET*, *MET-LINC01392*, *LRIG3-MET*, *PRKAR1A-MET, STARD3NL-MET*, *MET-STEAP4*, *TFEC-MET*, *PRKAR2B-MET*, *TRIM4-MET*, *THAP5-MET*, *TNPO3-MET*, *MET-UBE2H*, *WEE2-AS1-MET*, *WNT2-MET* and *LINC01392-MET*, are found in sporadic cases, and each type was detected in only one patient (1/74, 1.4%). Compared with the concomitant *MET* exon 14 skipping mutation (2/60, 3.3%) among the included patients, concomitant *MET* amplification (16/69, 23.2%) seemed to be more strongly related to *MET* fusions, consistent with the results of in vitro experiments in previous research [19]. The genes encoding *MET* fusion partners are distributed on various chromosomes, but most of them are located on the same chromosome as the *MET* gene [18].

We then explored the comutation patterns of patients harboring *MET* fusions in the cBioPortal for Cancer Genomics database as shown in Fig. 1C; a total of 16 genes, namely, *EGFR*, *MDM2*, *RBM10*, *CDK4*, *GLI1*, *PIK3C2G*, *EPHA7*, *DOT1L*, *CAPZA2*, *LRP1B*, *CDK6*, *MYCL*, *PNRC1*, *EPHA2*, *NRG3* and *COMETT*, were found to have statistically significant (*P* < 0.05) comutation rates with *MET* fusions. In a particular research work [19], scientists also documented the mutation features found in patients with *MET* fusions. The proposal included the co-mutation of CDK6 and RBM10 with MET fusions, a finding that was also affirmed in the recent review. However, the underlying connections between the mutations and the functional changes in the proteins encoded by these genes remain to be further elucidated.

### Treatment for patients harboring *MET* fusions: therapeutic options and efficacy of MET-TKIs

According to previous studies and reported cases, as shown in Table 1, we found that multiple MET-TKIs were proven to be effective in patients harboring *MET* fusions. Seven kinds of MET-TKIs, including crizotinib, cabozantinib, tepotinib, capmatinib, savolitinib, PLB-1001 (bozitinib) and ensartinib, were reported in the treatment of patients harboring *MET* fusions. The best therapeutic response status was also considered in this review.

Crizotinib was reported in the treatment of 22 patients with *MET* fusions: as first-line treatment in 8 patients and as postfirst-line treatment in 14 patients. Crizotinib demonstrated dramatic efficacy in patients harboring *MET* fusions, as listed in Table 1. Four patients who received cabozantinib were reported. However, only 1 patient received first-line cabozantinib treatment, and PFS was not reached (NR) as reported. Four patients had received tepotinib treatment, with one of them experiencing a complete response (CR), two showing a partial response (PR), and one having progressive disease (PD) as the best response. The remaining 3 patients received post first-line cabozantinib treatment; one of them showed a PR to the treatment. Another 3 patients who received capmatinib were reported, and all three patients achieved PR. Fewer than three patients each received treatment with tepotinib, savolitinib, PLB-1001 (bozitinib) and ensartinib.

As summarized earlier in this review, aberrant fusions of *MET* can enhance the expression and lead to abnormal activation of the MET protein, indicating that MET-TKIs have the potential ability to inhibit activated MET proteins and the downstream signaling pathways induced by these *MET* fusions. The evidence for crizotinib in the treatment of patients harboring *MET* fusions is currently extensive; the efficacy of crizotinib is satisfactory, and the safety is tolerable. Patients harboring a series of MET fusion types, including *CAV1-MET*, *EHBP1-MET*, *ARL1-MET*, *CUX1-MET*, *MET-DSTN*, *CD47-MET*, *HLA-DRB1-MET*, *STARD3NL-MET*, *MET-ATXN7L1*, *MET-UBE2H*, *EPHB4-MET* and *CCDC6-MET*, responded well to crizotinib treatment. Patients harboring these types of MET fusions achieved PRs and even CRs. Patients harboring *CD74-MET* and *KIF5B-MET* demonstrated different responses to crizotinib treatment, as listed in Table 1. One patient harboring *TNPO3-MET* did not benefit from crizotinib treatment, even as the first-line treatment. For patients harboring *CD47-MET* and *HLA-DRB1- MET*, the efficacy of MET-TKIs seems to be certain, and all patients showed a response to MET-TKI treatment. However, the efficacy of different MET-TKIs in patients harboring *MET* fusions must be further verified, given that the incidence of *MET* fusions is too low to draw any robust conclusions. However, we still recommend crizotinib as a potentially suitable choice for patients harboring *MET* fusions, regardless of the treatment line. It is worth noting that the clinical results of MET fusion-positive patients treated with MET-TKIs were aggregated from case reports and retrospective research. In addition, the mechanisms behind the response to MET-TKIs in patients with MET fusions are still not well understood. If the kinase domain of MET was not included in the fusion protein, these patients harboring related fusion genes naturally had no response to MET-TKI treatment. The functions of *MET* fusions with distinctive fusion partners or fusion sites and their sensitivity to targeted drugs require further research.

### Characteristics and clinical importance of primary and acquired *MET* fusions in malignancies

According to published studies in related fields, it is believed that abnormal expression or activation of the MET protein is tightly associated with the development of TKI resistance. The promotion of MET expression serves as the major bypass mechanism involved in resistance to EGFR-TKIs [39]. Abnormal MET protein expression could not only activate the classical downstream MAPK pathway, Akt pathway and STAT3 pathway, all of which participate in the inhibition of apoptosis induced by TKIs as concluded above, but also activate the MET/MYC/AXL axis and enhance resistance [40–42]. Given the critical role of acquired abnormal MET function in inducing resistance to TKIs, a previous study also shed new light on strategies for combination therapy, including therapies combining relevant TKIs and MET inhibitors (MET-TKIs), in improving the prognosis of patients [32, 43]. As we summarized above, aberrant fusion of the *MET* gene could act as a trigger for the upregulation and activation of MET. Thus, mechanistically, MET inhibition could be an underlying method for salvage therapy for patients harboring *MET* fusions.

In this review, a total of 39 patients with primary *MET* fusions detected and 9 patients (4 of whom had detailed treatment information) with acquired *MET* fusions detected were included as shown in Table 1. According to the summary of the 4 patients harboring acquired *MET* fusions, we found that treatment with a MET-TKI alone or in combination with TKIs relevant to the primary targets, such as the EGFR-sensitive mutations in these patients, achieved promising efficacy in these patients after the development of resistance to TKIs relevant to the primary targets. Seventy-five percent of these 4 patients (3/4) achieved a PR after treatment, and 1 patient achieved a CR. The median PFS time of patients treated with the combination of MET-TKIs plus EGFR-TKIs was NR by the time of publication of these reports. Importantly, 2 of the patients harboring acquired *MET* fusions were administered crizotinib plus EGFR-TKIs (icotinib or gefitinib) as fourth-line treatment but still exhibited dramatic responses to the treatment and achieved a PR and even a CR. Therefore, combination therapy is supposed to be an efficient salvage treatment for patients who develop resistance to TKIs relevant to the primary targets. The latest study [18] compared the genes encoding *MET* partners for primary and acquired *MET* fusions, and the findings suggested a significant difference in the functional enrichment of the genes between the two groups. Interestingly, MET fusions correlated with a lower tumor mutation burden as illustrated in Fig. 1D.

## Conclusion

MET fusions could be targetable genomic variants of *MET*, and inhibition of MET is considered the baseline therapeutic choice for patients harboring *MET* fusions. According to the summary presented in this review, we recommend MET-TKIs, especially crizotinib, as suitable agents for the treatment of patients harboring primary *MET* fusions. For patients harboring acquired *MET* fusions after the development of resistance to TKIs targeting primary genomic alterations, such as sensitive EGFR mutations, treatment with a MET-TKI alone or in combination with TKIs targeting primary genomic alterations, such as EGFR-TKIs, is hypothesized to be a reasonable option for salvage treatment. In summary, *MET* fusions, despite their low incidence, should be taken into consideration when developing treatment strategies for cancer patients.

## Data Availability

All data and material from this study are available.

## Abbreviations

TKIs: tyrosine kinase inhibitors
EGFR: epidermal growth factor receptor
PFS: progression-free survival
OS: overall survival
NSCLC: non-small cell lung cancer
ALK: anaplastic lymphoma kinase
NTRK: neurotrophin receptor kinase
HGF: hepatocyte growth factor
LUAD: lung adenocarcinoma
NGS: next-generation sequencing
FFPE: formalin-fixed paraffin-embedded
PR: partial response
CR: complete response
NR: not reached
PD: progressive disease
